# Understanding the Biology of Self-Renewing Macrophages

**DOI:** 10.3390/cells7080103

**Published:** 2018-08-09

**Authors:** Tamás Röszer

**Affiliations:** Institute of Neurobiology, University of Ulm, Albert-Einstein-Allee 11, 89081 Ulm, Germany; tamas.roeszer@uni-ulm.de

**Keywords:** macrophage, leukocyte biology, stem cell, proliferation

## Abstract

Macrophages reside in specific territories in organs, where they contribute to the development, homeostasis, and repair of tissues. Recent work has shown that the size of tissue macrophage populations has an impact on tissue functions and is determined by the balance between replenishment and elimination. Macrophage replenishment is mainly due to self-renewal of macrophages, with a secondary contribution from blood monocytes. Self-renewal is a recently discovered trait of macrophages, which can have a major impact on their physiological functions and hence on the wellbeing of the organism. In this review, I discuss our current understanding of the developmental origin of self-renewing macrophages and the mechanisms used to maintain a physiologically stable macrophage pool.

## 1. Introduction

Macrophages have long been considered terminally differentiated immune cells that develop from monocytes and are unable to enter the cell cycle [1,2,3]. Recent studies, however, challenge this view and propose that macrophages can indeed enter the cell cycle and self-renew [2,4]. Moreover, macrophages can develop from embryonic leukocyte precursors without the need for a monocyte intermediate, as has been illustrated for some tissue-resident macrophage pools such as the microglia [5]. Bone marrow-derived, hence monocyte-derived, macrophages can infiltrate most tissues, where they contribute to the maintenance of the macrophage pool [2]. Skin and intestinal mucosa, for instance, are colonized mainly by monocyte-derived macrophages [6,7]. Other tissues harbor macrophages derived from embryonic progenitors and monocytes to varying degrees [2].

**Figure 1:**
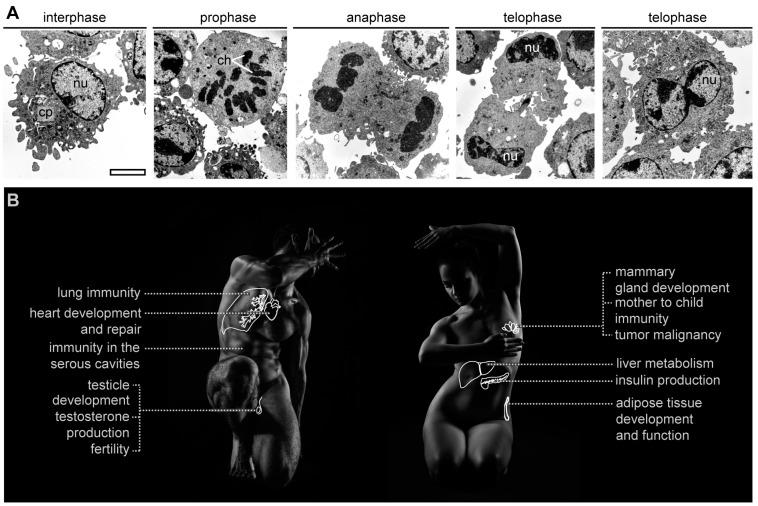
Self-renewing macrophages are key for organ development and function. (**A**) Transmission electron microscopy images of macrophages in the cell cycle. cp: cytoplasm, nu: nucleus, ch: chromosomes, scale bar 10 μm; (**B**) Summary of developmental, repair, metabolic, and immune functions that are dependent on self-renewing macrophages.

Tissue-resident macrophages are long-lived and, as shown by recent findings, can self-renew (Figure 1A). However, we still lack a full empirical understanding of the developmental program of various tissue-resident macrophages, the microenvironmental cues that signal their self-renewal, and the proportion of embryonic- or monocyte-derived macrophages in tissues. Also, it is still a subject of research whether tissue-resident macrophages self-renew in the entire life span or during development only. The available data suggest that developing and maintaining appropriately sized and physiologically beneficial tissue-resident macrophage pools has an impact on health (Figure 1B).

**Figure 2:**
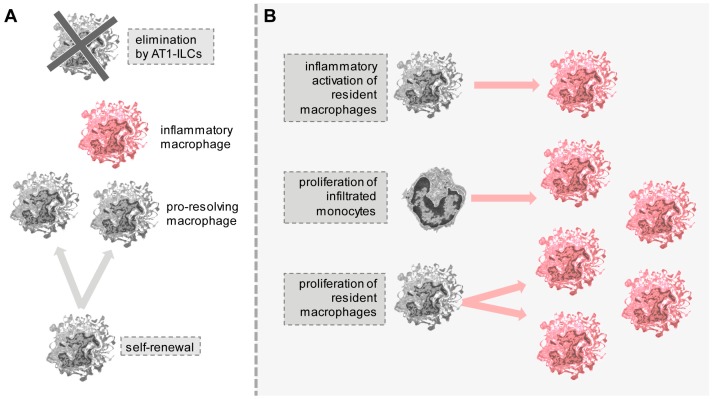
An example of balanced macrophage replenishment and elimination. (**A**) Under physiological conditions, macrophages of the adipose tissue are replenished by self-renewal, and a monocyte contribution is not necessary to build the macrophage pool; the majority of the macrophages are naïve or pro-resolving. Excess macrophages are removed by adipose tissue type 1 innate lymphoid cells (AT1-ILCs); (**B**) In obesity, macrophage number increases via immigrating monocytes and excessive macrophage proliferation, and the macrophages are predominantly inflammatory.

The importance of a balanced self-renewal and removal of macrophages is exemplified by the resident macrophage pool of the adipose tissue. Replenishment is supported by local self-renewal [8], while the elimination of excess macrophages is accomplished by type 1 lymphoid cells [9] (Figure 2A). These antagonistic mechanisms ensure that macrophages do not induce unwanted fibrosis and vasculogenesis or exaggerated inflammation, which would negatively affect adipose tissue physiology [10]. Macrophages comprise approximately 10% of all cells in the adipose tissue under physiological homeostasis, which can increase to 50% in obesity [11], when the balance between replenishment and removal of macrophages is impaired (Figure 2B). This can lead to the development of metabolic diseases such as insulin resistance, adipose tissue fibrosis, and diabetes, as a result of inflammation and extracellular matrix remodeling caused by excess macrophage number [10,12,13,14].

This example highlights that maintenance of tissue-resident macrophages impacts tissue function. In the light of recent findings discussed herein, it is very likely that rather than their replenishment from monocytes, self-renewal is a key mechanism that sustains the physiological macrophage pools in most organs. A better understanding of macrophage self-renewal is hence a path towards new approaches to manipulate tissue macrophages. In this review, I provide an up-to-date summary of the biology of macrophage self-renewal and the mechanisms that tissues use to dictate macrophage number.

## 2. Origin of Self-Renewing Tissue-Resident Macrophages

Long-lived tissue-resident macrophages can develop from embryonic hematopoiesis [2,15,16,17], although bone marrow-derived, hence monocyte-derived, macrophages can also infiltrate most tissues and contribute to the maintenance of the macrophage pool [2,18,19]. Microglia are generated from embryonic progenitors [5,20], whereas the skin and intestinal mucosa, for instance, are colonized mainly by monocyte-derived macrophages [6,7]. Other tissues harbor macrophages derived from both embryonic progenitors and monocytes to varying degrees [2].

In the mouse, the early embryonic progenitors of long-lived tissue-resident macrophages develop from endothelia-derived erythro-myeloid progenitors of the yolk sac [17,21,22]. This developmental program may be evolutionarily conserved, as suggested by studies of tissue macrophage development in amphibia. In this case, early macrophage progenitors develop from the anterior ventral blood island, which is the equivalent of yolk sac hematopoietic cells [23]. The development of the yolk sac-derived macrophage lineage in the mouse is Myb-independent, whereas monocyte-derived macrophages develop in a Myb-dependent manner [5]. Yolk sac erythro-myeloid progenitors can generate macrophages without a monocyte intermediate, but they also colonize the fetal liver where they give rise to Myb-expressing fetal monocytes [17]. In turn, these fetal monocytes can differentiate into tissue-resident macrophages [17]. There is, however, a paucity of information on the development of tissue-resident macrophages in humans. The human embryo yolk sac also generates CX_3_CR1^+^ macrophages, similarly to its mouse counterpart [24,25]. It has been demonstrated recently that human induced pluripotent stem cells differentiate into macrophages in a MYB-independent manner, resembling the developmental program of the murine yolk sac-derived macrophages [26]. These MYB-independent macrophage progenitors can further differentiate into tissue-resident macrophages once they are exposed to tissue-specific signals [27].

In a simplified view, yolk sac-derived tissue macrophages are CD11b^+^ F480^+^ CX_3_CR1^+^ CCR2^−^ cells with self-renewal ability. By contrast, monocyte-derived macrophages are CD11b^+^ F4/80^+^ CX_3_CR1^−^ CCR2^+^ [28]. There is evidence showing that monocyte-derived macrophages can also regain their proliferation ability, which can expand the tissue macrophage pool, as detailed in this review. There exist also organ-specific features of the ontogenetically distinct macrophages and their self-renewal mechanisms, which are further detailed below.

## 3. Self-Renewal Ability of Kupffer Cells

Liver-resident macrophages or Kupffer cells constitute the major resident macrophage population in the human body. Several studies have shown that the quantity of Kupffer cells has an impact on liver function. Kupffer cell number is restored to its initial level after Kupffer cell depletion [29,30]. Also, a high Kupffer cell number increases patient survival rates following liver transplantation [31], and alterations in Kupffer cell number are associated with specific liver pathologies [32], although distinguishing bona fide Kupffer cells from infiltrating monocytes and monocyte-derived macrophages is challenging. Akin to other macrophage populations, Kupffer cells were long believed to lack the ability to proliferate [33], and monocytes were thought to be their sole source for replenishment [34]. However, recent lineage-tracing studies of myeloid cell development have questioned this view and have showed that under homeostatic conditions Kupffer cells are maintained by self-renewal without the contribution of blood monocytes [5,35]. Indeed, embryonic development of macrophages and monocytes is distinct. Analogous to many other tissue-resident macrophages, Kupffer cells develop from yolk sac-derived macrophage progenitors [2], which enter the blood stream and seed the fetal liver where they retain a high proliferative capacity. These self-renewing macrophages further mature into Kupffer cells [5].

The proliferation of Kupffer cells is macrophage colony stimulating factor (M-CSF)-dependent, as demonstrated by the reduced number and immature morphology of Kupffer cells in osteopetrotic (op/op) mice, which lack functional M-CSF. Replacement of M-CSF in these mice restores Kupffer cell proliferation and maturation [36]. Similarly, in neonatal rats, M-CSF increases Kupffer cell number without affecting blood monocyte count [37]. Other signals, such as erythropoietin, have also been shown to increase Kupffer cell proliferation [38].

Similar to what found in the steady state, the Kupffer cell population can be restored by self-renewal following liver injury without the need for blood monocytes [39]. For instance, paracetamol overdose leads to Kupffer cell repopulation solely by proliferation [39]. However, the niche of Kupffer cells can be occupied by monocyte-derived macrophages after Kupffer cell death in *Listeria monocytogenes* infection [40]. Monocyte-derived macrophages, which replace Kupffer cells, are also capable of self-renewal [18,29]. In some instances, however, monocyte-derived Kupffer cells lack self-renewal ability [41]. Other transient macrophage types are involved during the recovery from liver injury, including Ly6C^high^ monocytes, which differentiate into Ly6C^low^ macrophages [39]. In response to erythropoietin, Kupffer cells increase their production of monocyte chemoattractant protein-1 (CCL2), which boosts the infiltration of Ly6C^high^ monocytes into the liver [38]. Monocyte-derived macrophages are nevertheless distinct from Kupffer cells. These immature cells are found in high amounts in the liver of obese mice, are more inflammatory than Kupffer cells, and contribute to the severity of liver injury in obesity [42]. Interestingly, by secreting CCL2, Kupffer cells increase the prevalence of monocyte-derived cells in the liver. These cells express the CCL2 receptor CCR2 at levels five-fold higher than Kupffer cells, which allows them to respond effectively to CCL2 [42]. Still, a recent mouse study showed that monocytes residing in the liver can also self-renew and replenish Kupffer cells [29]. It has also been observed that the Y chromosome of male bone marrow donors appears in Kupffer cells of female recipients, suggesting that the bone marrow also contributes to Kupffer cell replenishment [34].

In summary, local proliferation of Kupffer cells is key for their homeostatic maintenance. In the setting of inflammation and tissue repair, however, bone marrow-derived macrophages can also establish in the liver, and these monocyte-derived cells have proliferation ability. Whether the developmental program and self-renewal control of these two self-renewing macrophage populations is distinct and can be modified by pharmacological intervention is unknown.

## 4. Self-Renewal of Lung Macrophages

The resident macrophages of the lung alveoli are called alveolar macrophages and were previously thought to be replenished from circulating monocytes in adulthood [43]. Local proliferation of alveolar macrophages has been reported in mice [44,45] and humans [46], and they have also been shown to retain their self-renewal capacity in vitro [46,47]. Recent studies suggest that alveolar macrophages develop from F4/80^high^ CD11b^low^ primitive macrophages and Ly6C^high^ CD11b^high^ fetal monocytes around embryonic day (E) 12.5–16.5 in mice, and the mature alveolar macrophages self-renew throughout the lifespan [48]. Bone marrow-derived monocytes also settle in the lung with aging [5] and in disease [49] and can be long-lived [49]. Mitogenic signals for alveolar macrophages include M-CSF and granulocyte/macrophage colony-stimulating factor (GM-CSF) [1], which are most likely provided by resident lung epithelia and fibroblasts [1,47,50,51], and also interleukin (IL)-1α [52]. Hyperoxia, which is a complication of respiratory support in preterm infants, reduces alveolar macrophage proliferation [53]. By contrast, pulmonary fibrosis and allergic inflammation increase alveolar macrophage number [44,47], which is due in part to monocyte-derived macrophage infiltration [44]. It is also possible that increased mitogenic signaling from pulmonary fibroblasts can bolster the local proliferation of alveolar macrophages [47]. Importantly, monocyte-derived macrophages promote allergic lung inflammation, whereas macrophages that are generated by self-renewal protect against inflammation [44]. Because alveolar macrophages can increase the extent of fibrosis and support tumor growth [54], their elevated number and increased self-renewal may be unfavorable in these settings.

## 5. Macrophage Self-Renewal in the Serous Cavities

Serous cavities are the pericardium, the pleural, and the peritoneal cavities. All have been found to be rich in resident macrophages [55]. During embryogenesis, these macrophage populations develop from yolk sac- and fetal liver monocyte-derived macrophages [1,2,35]. Pleural macrophages have been reported to self-renew under physiological conditions [56,57]. Similarly, at least one subpopulation of peritoneal macrophages, which are yolk sac-derived F4/80^high^ GATA6^+^, are long-lived, undergo self-renewal and maintain their population for at least four months in mice. However, as is observed in other yolk sac-derived tissue-resident macrophages, these peritoneal macrophages are gradually replaced by monocyte-derived macrophages [18,56]. Peritoneal macrophages can also arise from the so-called milky spots of the omentum [58], which are immune aggregates comprising monoblasts [59,60,61] and are thus capable of generating myeloid cells [58]. Milky spots develop from the 20th week of gestation in humans and provide a microenvironment that allows homing and local proliferation of macrophage precursors [58,60].

Whether there is a clear difference between the function of yolk sac-derived and monocyte-derived macrophages in the serous cavities is unclear. In the peritoneum, yolk sac-derived macrophages may be the equivalent of the so-called large peritoneal macrophages (LPMs), a macrophage subset classified according to their morphology. Conversely, the so-called small peritoneal macrophages (SPMs) are believed to develop from bone marrow progenitors [62]. There may indeed be functional differences between these populations in the peritoneum. For example, LPMs, which are the most abundant subset in steady-state conditions, express the canonical markers CD11b and F4/80 at levels approximately three times and 80 times higher, respectively, than SPMs. Moreover, LPMs express the surface markers Gr-1 and AA4.1, which are absent from SPMs. In addition, CD40, CD80, CD86, CD11c, and TLR4 are all expressed at higher levels in LPMs than in SPMs. By contrast, SPMs are MHC-II^+^, whereas LPMs are MHC-II^−^. Phagocytosis and nitric oxide production of these subsets may also differ, but the reported differences depend on the experimental conditions [62]. Furthermore, IL-4 responsiveness of yolk sac- and monocyte-derived macrophages is distinct. Accordingly, only monocyte-derived macrophages upregulate retinaldehyde dehydrogenase 2 and programmed cell death 1 ligand 2 in response to IL-4 [16]. Monocyte-derived macrophages also have high levels of aldehyde dehydrogenase activity and produce retinoic acid, whereas locally generated macrophages express high levels of mitochondrial uncoupling protein 1 [16].

## 6. Self-Renewing Resident Macrophages of the Heart and the Artery Wall

Macrophages in the heart have key roles both in cardiac development and remodeling, and a very recent study proposed that they are also necessary for electrical conductance [63,64]. In mice, cardiac macrophages appear at E10.5 and can be classified into various subsets, such as yolk sac-derived CCR2^−^ and CCR2^+^ macrophages, recombination activating gene 1-positive macrophages, which are lympho-myeloid cell-derived, and Fms-like tyrosine kinase 3-positive macrophages, which develop from fetal monocytes [65]. Macrophage precursors have been shown to seed the embryonic heart beneath the epicardium [66]. With aging, these embryonic, self-renewing macrophages are gradually infiltrated by monocyte-derived macrophages [67,68].

We still know little about the specific functions of macrophage self-renewal in the heart. It has been shown, however, that coronary artery morphogenesis requires yolk sac-derived CCR2^−^ heart-resident macrophages [65] and, in adulthood, a local expansion of cardiac macrophages occurs during cardiac remodeling [63]. Moreover, a recent study showed that cardiac macrophage proliferation occurs within the first week following pressure overload hypertrophy and is necessary for the myocardial adaptive response [69].

The adventitial layer of the large arteries has also been found to contain resident macrophages that develop from CX_3_CR1^+^ yolk sac macrophage precursors and retain self-renewal ability [70]. In adults, these arterial macrophages are sustained by local proliferation. Moreover, because arteries contain mesenchymal cells that produce the CX_3_CR1 ligand CX_3_CL1, the CX_3_CR1^+^ resident macrophage pool can be maintained [71].

## 7. Self-Renewal of Pancreatic Macrophages

Recently, it has been shown that the pancreatic islets harbor long-lived macrophages, which express CD11c, MHC-II, F4/80, CD11b, CD64, lysozyme, CX_3_CR1, and CD68 [72]. Resident macrophages of the pancreatic islets are in contact with blood vessels and β cells, control the endocrine function of the islet [73,74], and sense microbial products from the blood [75]. In a parabiosis experiment, the pancreatic islet-resident macrophages had negligible replacement by non-host-derived monocytes [72]. When bromodeoxyuridine (BrdU) was used to measure cell proliferation, 20–50% of the islet-resident macrophages incorporated BrdU within approximately seven days [72]. These findings show that islet-resident macrophages are long lived, self-renew, and are not replaced by circulating monocytes under homeostatic conditions.

The exocrine pancreas contains a CD206^+^ macrophage subset which develops from yolk sac macrophage progenitors and fetal liver monocytes, and a second, CD206^−^ subset which is replenished from bone marrow-derived monocytes [72]. Those macrophages, which develop from yolk sac macrophage progenitors and fetal liver monocytes, show a lower self-renewal rate than the islet-resident macrophages, although they are still maintained without the need of bone marrow-derived monocytes [72]. Altogether, at least two resident macrophage pools of the pancreas have a long half-life and are maintained by self-renewal. Following whole-body lethal irradiation, however, the empty niches of both macrophage pools are refilled with bone marrow-derived macrophages [72].

The health impact of the self-renewal ability of these two macrophage populations is still to be determined. Islet-resident macrophages present antigens, express genes associated with inflammatory macrophage activation, and are involved in the development of autoimmunity against pancreatic β cells [72,75]. Hence, the renewal of this macrophage pool may impact the inflammatory state in the islets. In line with this, lack of M-CSF or neutralization of M-CSF by an antibody result in the decrease of the pancreatic resident-macrophage pool, which has been shown to reduce the development of autoimmune diabetes [72,73]. Moreover, pancreatic islet development requires the presence of locally proliferating CCR2^+^ macrophages within the neonatal pancreas in mice [76]. The lack of the locally proliferating CCR2^+^ macrophages leads to a reduction in β cell proliferation, dysfunctional islet phenotypes, and glucose intolerance in newborns [76]. CCR2^+^ myeloid cells are prominent sources of IGF2, which contributes to IGF1R-mediated islet proliferation [76]. This makes plausible that the establishment of the pancreatic macrophage pools requires self-renewal ability of resident macrophages, and this is needed for healthy islet function.

## 8. Self-Renewal of Adipose Tissue Macrophages

Adipose tissue is a reservoir for innate immune cells [77], the most prevalent of which are resident adipose tissue macrophages (ATMs), whose functions are to dispose of dying adipocytes, remove cellular pathogens, process lipids, and release various mediators that maintain a healthy metabolism [10,78]. Accordingly, adipose tissue is critically dependent on resident ATMs to perform these vital tasks [10,79,80,81]. ATMs develop from yolk sac progenitors, but monocytes also contribute to the ATM pool [12,35,82]. Indeed, bone marrow transplantation cannot replace the entire ATM pool in the mouse [83]. This dual origin of ATMs may explain why some metabolic effects of ATMs cannot be transmitted by bone marrow transplantation [84].

The metabolic status of adipocytes is a determining factor of ATM output. Under homeostatic conditions, ATMs adopt a pro-resolving phenotype [81] and provide metabolic benefits by supporting the function of adipocytes; however, owing to their roles in tissue remodeling and injury response, they can provoke adipose tissue fibrosis by expressing pro-fibrotic mediators [85], which may be unfavorable. As mentioned earlier, recent studies have shown that there are balanced replenishment and removal of pro-resolving ATMs, which function to maintain a metabolically beneficial ATM pool [8,9]. ATM homeostasis is believed to be sustained by self-renewal [8] and not to depend on circulating monocytes [83]. The adipose tissue also has a mechanism to eliminate ATMs via the activity of type 1 innate lymphoid cells [9] (Figure 2A). In obesity, this balance is compromised, increasing the total ATM number and provoking the dominance of inflammatory ATMs [8,12,86] (Figure 2B).

Specifically, in obese adipose tissue, lipid-overloaded adipocytes undergo apoptosis or secondary necrosis, releasing various danger signals such as modified lipids and nucleic acids [78]. In response to this, ATMs adopt an inflammatory activation state that is damaging to the metabolism [10] and secrete inflammatory mediators and nitric oxide, resembling a pathogen-killing immune response [80,81]. These inflammatory mediators inhibit insulin signaling, which in turn provokes insulin resistance in the adipose tissue. When adipose tissue inflammation persists for a prolonged period, ATM-derived inflammatory mediators are released into circulation, leading to the development of systemic insulin resistance [10,87] and also driving type 1 diabetes mellitus via secondary toxic effects on the pancreatic β cells [87]. Obesity increases ATM self-renewal, giving rise to an increase in the number of inflammatory ATMs [12,88,89]. Moreover, obesity boosts monocyte infiltration of the adipose tissue, and ATMs increase myelopoiesis, accelerating monocyte infiltration into the obese adipose tissue [90,91]. Impeding inflammatory ATM expansion in the obese adipose tissue and increasing the amount of pro-resolving resident ATMs are envisaged as treatment modalities for insulin resistance [10,78]. However, the metabolic impact of the pro-resolving ATMs is still not fully understood [92,93], and excess number of pro-resolving ATMs also can have unwanted metabolic effects [14]. Moreover, it is also an intriguing question whether long-lived tissue-resident ATMs give rise to inflammatory ATMs in obesity, or the resident ATMs are replaced by monocyte-derived inflammatory macrophages [94]. Similarly, it is still not well defined whether, upon the resolution of inflammation, the inflammatory ATMs adopt a pro-resolving phenotype. This possibility is suggested by in vitro studies showing that the inflammatory macrophage phenotype can be reverted to a pro-resolving phenotype [81,95]. It is also possible, however, that pro-resolving resident ATMs expand and restore tissue homeostasis [8]. However, today we lack pharmacological tools which would effectively alter the inflammatory/pro-resolving ATM phenotype without inducing negative effects on the innate immune response [10,96]. Moreover, the lack of an effective “switch-off mechanism” of cytotoxic nitric oxide secretion by inflammatory macrophages challenges the possibility that an inflammatory macrophage is able to regain a pro-resolving phenotype (this possibility is further discussed in [97]). While it remains to be seen how ATM self-renewal is supported under physiological conditions and in obesity, it is possible that cell cycle entry of pro-resolving and pro-inflammatory ATMs is controlled by distinct signaling mechanisms.

## 9. Self-Renewal of Resident Macrophages in the Mammary Gland

The mammary gland is rich in resident macrophages, which accumulate in the vicinity of the gland ducts [98]. A major function of mammary gland macrophages is the organization and remodeling of the extracellular matrix during development, in addition to their roles in the removal of apoptotic epithelial cells during gland morphogenesis [98]. In the mouse, mammary gland macrophages are F4/80^+^ CD11b^+^, similar to other tissue-resident macrophages [98], whereas human mammary gland-resident macrophages express CD68 [99]. With the exception of macrophages and eosinophils, other leukocyte types are absent from the mammary gland except during lactation, when lymphocytes are abundant and secrete maternal immunoglobulins into the breast milk [98]. Along this line, it has been reported that leukocytes are important for mammary gland development, as their absence leads to a neonatal gland morphology in mouse [98].

Macrophages are also present in breast milk, and while their precise immunological impact remains uncertain, they seem to have roles in disease transmission from mother to child [100,101]. Breast milk macrophages show unique features that distinguish them from the resident macrophages of the mammary gland. For example, human breast milk macrophages produce GM-CSF spontaneously and differentiate into CD1^+^ cells—thought to be dendritic cells—in response to IL-4 [101]. These unique functional features are likely to be induced specifically by phagocytosis of breast milk components [101]. Moreover, unlike resident macrophages of the mammary gland, breast milk macrophages express DC-SIGN, a dendritic cell-specific lectin that mediates interactions with the human immunodeficiency virus (HIV). Accordingly, breast milk macrophages can be involved in the transmission of HIV during breast feeding [101].

While the lineage of mammary gland macrophages remains elusive, they appear around the so-called terminal end bud (TEB) structure, which is observed during the early stages of mammary gland development [102]. Macrophages surround the developing neck of the TEB, and this pattern of distribution distinguishes them from eosinophils, which are also frequent leukocytes in the mammary gland [98].

Macrophage numbers have been reported to increase both in benign and malignant human breast tumor tissue, with significantly higher numbers found in malignant tumors [103]. Tumor-associated macrophages have features that discriminate them from the resident macrophages of the mammary gland. In mouse models of spontaneous breast cancer, CD11b^low^ CD206^+^ MHC-II^low^ macrophages are present that are capable of self-renewal via M-CSF-dependent proliferation [104]. Signaling from gland cell epithelia to macrophages through M-CSF is necessary for the normal development of the mammary gland [97]; however, M-CSF is a mitogenic signal for macrophages, and, hence, uncontrolled M-CSF signaling can increase macrophage proliferation in the breast. An increase in the macrophage content of the mammary gland can promote tumor growth and metastasis by various mechanisms, such as vascularization promotion, matrix remodeling, and epithelial adhesion reduction [105]. CCL2 produced by cancer cells and myeloid cells attracts CD206^+^ Tie2^+^ macrophages into the breast tissue, further increasing the macrophage content of the breast [104].

Macrophage number is crucial both for the normal development of the breast and for tissue homeostasis: as mentioned earlier, the absence of macrophages retains the mammary gland in an immature state and also impedes tumor growth. The malignant behavior of cancer cells can be regulated by their microenvironment, which in turn is largely affected by macrophage number and function [106]. Hence, factors that control the local expansion of mammary gland-associated macrophages have medical potential. To date, however, limited data are available about the physiological regulation of mammary gland macrophage development and self-renewal.

## 10. Self-Renewal of Testicular Macrophages

Testicular macrophages are the largest leukocyte population in the testis and play essential roles in fetal testicular development and homeostasis in adults [107]. Following puberty, when spermatogenesis is initiated, sperm-specific neoantigens are expressed in the testicle, and this setting requires a mechanism of self-tolerance to these antigens. Testicular macrophages contribute to self-tolerance through the production of immunosuppressive cytokines, such as IL-10 and TGF-β, and by low expression of proinflammatory cytokines [108]. In addition, testicular macrophages are important for testosterone synthesis due to their interactions with Leydig cells, which are epithelioid cells that produce testosterone. Two distinct populations of macrophages can be found in the adult testis on the basis of their localization and embryonic origin: an interstitial macrophage population, which develops from embryonic macrophage progenitors and maintains immunological self-tolerance and aids testosterone production, and a peritubular macrophage population, which develops postnatally in the prepuberty period from definitive bone marrow hematopoietic progenitors [109]. The peritubular macrophage population is associated with spermatogonial tubes in proximity to spermatogonial stem cells and has a potential role in their differentiation [109].

Testicle-resident macrophages are Ly6C^−^ CD11c^−^ F4/80^+^ CD11b^+^ CX_3_CR1^+^. The two macrophage populations have distinctive features when analyzed by flow cytometry [109]. Accordingly, interstitial macrophages are M-CSFR^+^ MHCII^−^ MerTK^+^ CD64^+^, whereas peritubular macrophages are M-CSFR^low^ MHCII^+^ MerTK^−^ CD64^−^ (for further details of the characterization of testicular macrophages, refer to [109]). The resident testicular macrophages also express CD163. Systemic inflammation in response to lipopolysaccharide (LPS) leads to an influx of CD163^−^ monocyte-like, infiltrating macrophages into the rodent testis that have a pro-inflammatory phenotype and express IL-1β, TNF-α, and inducible nitric oxide synthase. Conversely, resident CD163^+^ macrophages constitutively produce IL-10 and contribute to testicular immunosuppression [110,111]

Once established, both the peritubular and interstitial macrophage populations exhibit a long life span and a low turnover in the steady state [109]. Proliferating macrophages give approximately 8% of the testicular macrophage population at birth in mouse, which declines to 0% in adulthood in mice [109]. Two weeks after birth, 3% of the macrophages derived from embryonic hematopoiesis are in the S-phase of the cell cycle. This percentage decreases to 0.5% at six weeks of age. However, as the proliferative capacity of interstitial macrophages declines, monocytes start to contribute to the testicular macrophage population. The macrophage population which emerges from the definitive bone marrow hematopoiesis occurs two weeks after birth in the mouse testicle and gives rise to the peritubular macrophage pool [109]. Once steadily established, both macrophage populations have a long life span and can be detected for even 24 weeks in the mouse testicles [109]. Testicular macrophage proliferation might be controlled by hormonal signals; however, this has been largely unexplored. During the onset of puberty, testicular macrophage content increases in rats, likely as a response to gonadotropin hormone [112,113]. Two subsets of testicular macrophages in rats have been identified on the basis of their expression of a surface antigen recognized by monoclonal antibody ED2: ED2 (CD163)^+^ (resident-type) and ED2 (CD163)^−^ (monocyte-like) macrophages [114]. The number of ED2^+^ resident macrophages is controlled by Leydig cells [114]. The endocrine disruptor bis(2-ethylhexyl) phthalate has been shown to increase the population of peritubular macrophages [115]. Phthalates are androgen antagonists [116] and are commonly used plasticizers in many consumer products, including toys [115]. Accordingly, understanding the signals that control testicular macrophage self-renewal is key as these macrophages can impact male hormone production and fertility.

## 11. Signal Mechanisms and Regulators of Macrophage Self-Renewal

M-CSF is considered the major signal essential for determining macrophage number, while IL-34 is important in some tissue macrophage populations such as microglia and skin Langerhans cells [7,36,37,117]. Both M-CSF and IL-34 signal through the M-CSF receptor on macrophages [7,36,37,117]. Interestingly, macrophages of the liver and the spleen specifically degrade M-CSF, reducing its local availability [118]. This might therefore be one determinant of the size of the macrophage pools in their specific tissue niches [37]. As detailed in this review, a proportion of tissue macrophages develop from bone marrow progenitors, and tissue macrophages may control bone marrow hematopoiesis via feedback mechanisms; for example, ATMs increase bone marrow myelopoiesis in obesity [90].

**Figure 3:**
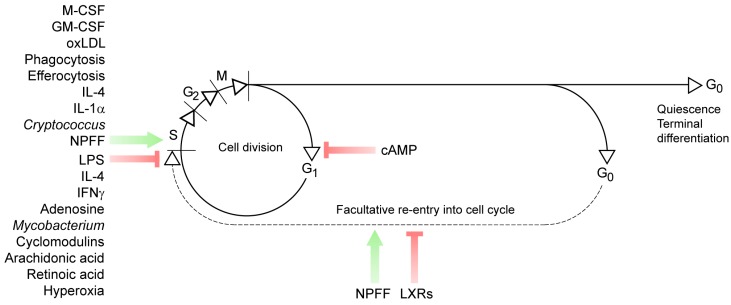
Signals affecting macrophage cell cycle. The scheme summarizes known signals that impede or facilitate cell cycle entry and cell cycle progression in macrophages. M-CSF: macrophage colony stimulating factor, GM-CSF: granulocyte/macrophage colony stimulating factor, oxLDL: oxidized low-density lipoprotein, LPS: lipopolysaccharide, NPFF: neuropeptide FF, LXRs: liver X receptors.

Little is known about the signals that increase cell cycle entry and sustain the self-renewal of tissue-resident macrophages (Figure 3). IL-4 seems to support macrophage self-renewal [13,57] and leads to M-CSF-independent proliferation of macrophages [119]. Nonetheless, it has also been shown that IL-4 inhibits M-CSF-dependent entry into the cell cycle [120]. We recently showed that neuropeptide FF (NPFF), which sustains IL-4 signaling in macrophages, increases macrophage self-renewal [8]. The underlying mechanism is, however, independent of IL-4 signaling and seems to be due to the suppression of cell cycle inhibitor genes such as the interferon inducible 200 family members [8]. Moreover, NPFF reduces the level of the transcription factor MafB in macrophages [8], and a decrease in MafB levels has been shown to increase the proliferation of macrophage progenitors [121,122]. In addition to NPFF, the nuclear receptor RXR (retinoid X receptor) is known to control MafB levels in macrophages [121]. Other signals, such as adiponectin [86], Fc-mediated phagocytosis and complement activation [123], uptake of *Cryptococcus* cells [123], and oxidized low-density lipoprotein (Ox-LDL) [124] are also known to increase macrophage self-renewal (Figure 3). The underlying mechanisms are not fully understood yet. For instance, it is known that Ox-LDL causes M-CSF release from macrophages, leading to p38 MAPK/Akt signaling, which may increase cell cycle entry of macrophages [125,126,127]. This effect of Ox-LDL is controlled by various signal mechanisms, such as those mediated by AMP-activated protein kinase [126] and NFκB [128].

Macrophages occupy specific niches in tissues, which raises the possibility that different tissue-resident macrophages have unique transcriptional programs to control self-renewal. For example, Kruppel-like factor 4 (KLF4) is key for regulating cardiac macrophage proliferation [69], mTORC2 signaling is necessary for peritoneal macrophage development [129], sirtuin-1 inhibition reduces alveolar and peritoneal macrophage self-renewal [130], and NPFF receptor expression is higher in ATMs than in other macrophage types [8]. Overall, these observations suggest that macrophage self-renewal may be controlled by tissue-specific mechanisms.

In the setting of inflammation, macrophage self-renewal is suspended, and macrophages adopt a pathogen-eliminating phenotype. This requires global transcriptional changes, for instance, a shift from Myc-controlled transcriptional control of self-renewal to a hypoxia-inducible factor alpha (HIF1α)-dependent transcriptional program for pathogen clearing. Inflammatory signals, hence, suppress Myc and also inhibit macrophage self-renewal [131]. It is well known that LPS, the major cell wall component of Gram-negative bacteria, arrests the macrophage cell cycle. The underlying mechanism involves the reduced expression of cyclin D1, which is key for cell cycle entry [132]. Moreover, LPS suppresses cyclin A2 expression and increases macrophage expression of cyclin-dependent kinase inhibitor 2D and nuclear factor of kappa light chain gene enhancer [133]. Overall, the effects of LPS inhibit the transition from G1 to S. Likewise, inflammatory mediators such as TNFα, IFNγ, and arachidonic acid, and intracellular pathogens such as *Mycobacterium* species block macrophage cell cycle progression [134,135,136,137,138]. The underlying mechanisms involve a reduced transcription of cell cycle “checkpoint” genes, which eventually block G1/S transition or slow down the cell cycle (Figure 3).

Despite its importance, there is a paucity of information on the signals that limit macrophage self-renewal in a physiological setting, which is important for tissue development and function. Nuclear receptor signaling, including liver X (LXR) and RXR, has been shown to block macrophage cell cycle [139,140], although many macrophages which have active LXR and RXR signaling can still enter the cell cycle [121,141]. Adenosine [142], cAMP [8,142,143], and the uptake of apoptotic cells [144] are also known to inhibit macrophage cell cycle (Figure 3). How these signals are orchestrated remains to be explored.

## 12. Concluding Remarks

**Figure 4:**
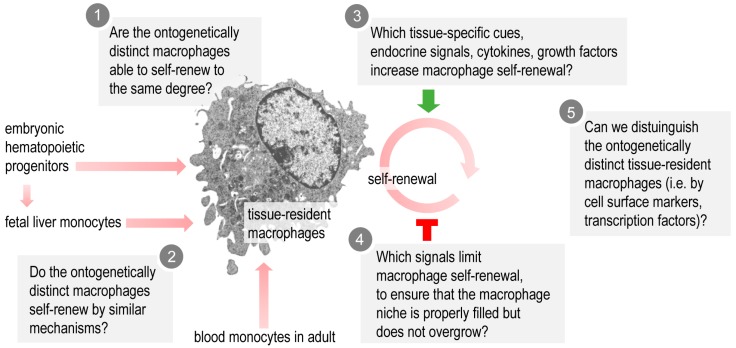
Open questions about tissue resident macrophage self-renewal. It is still unknown whether ontogenetically distinct tissue-resident macrophages have similar or distinct self-renew abilities (**1**); Answering this question will be important for determining the proportion of macrophages developing from embryonic or adult hematopoietic progenitors. It is not known whether ontogenetically distinct macrophages have their own specific mechanisms that allow self-renewal or induce quiescence (**2**); Tissue macrophage pools are stable in the physiological steady state; however, little is known about the signals that allow the build-up and replenishment of the macrophage pool by self-renewal (**3**); or limit the expansion of the macrophage niche (**4**); We still lack information about possible surface markers or transcription factors that clearly distinguish self-renewing macrophages (**5**).

Macrophage self-renewal is key for normal organ development and function. Accordingly, understanding macrophage self-renewal is potentially of high biomedical impact, since the macrophage number in tissues is key in certain diseases, such as insulin resistance, allergic inflammation, and fibrosis [44,145,146]. Lineage tracing of macrophage progenitors and the generation of chimeric animals with transplantation of macrophage progenitors are exciting new approaches to define tissue-specific mechanisms of macrophage self-renewal. However, we still lack a clear understanding of how macrophage self-renewal is controlled under physiological conditions (Figure 4). As reviewed here, tissue-derived cues, signals of the neuroendocrine system, energy status, bioactive molecules from the diet, as well as pathogen-associated signals, are all potential factors that control macrophage self-renewal. For instance, LXRs are lipid-controlled transcription factors [121], plasma NPFF levels are affected by the nutritional state [8], and the tissue levels of adenosine are directly linked to energy demand and circadian rhythmicity [147]. It is therefore possible that macrophage self-renewal is orchestrated by metabolic and nutritional signals. Deciphering these mechanisms should allow the dynamic control of macrophage pools and hence the prevention and treatment of inflammatory and metabolic diseases.

## Abbreviations

AA4.1	CD93, a C-type lectin-like protein
CCR2	C-C chemokine receptor type 2
CD11b	cluster of differentiation 11b
CD11c	cluster of differentiation 11c
CX_3_CR1	CX3C chemokine receptor 1
DC-SIGN	dendritic cell-specific ICAM-grabbing non-integrin
F4/80 antigen	EGF-like module-containing mucin-like hormone receptor-like 1
GATA6	GATA-binding factor 6
GM-CSF	granulocyte/macrophage colony stimulating factor
Gr-1	granulocyte receptor-1 antigen
Ly6c	GPI-anchored glycoprotein, which is expressed on a range of hematopoietic cell lineages
MafB	V-maf musculoaponeurotic fibrosarcoma oncogene homolog B
M-CSF	macrophage colony stimulating factor
MHC-II	major histocompatibility complex-II
mTORC2	mammalian target of rapamycin (mTOR) complex 2
Myc	mouse MYC proto-oncogene
MYC	human MYC proto-oncogene
NFκB	nuclear factor kappa-light-chain-enhancer of activated B cells
Tie2	angiopoietin receptor-2
TLR4	Toll-like receptor 4
